# Computational purification of tumor gene expression data

**DOI:** 10.1186/1471-2105-12-S11-A9

**Published:** 2011-11-21

**Authors:** Amit Deshwar, Gerald Quon, Quaid Morris

**Affiliations:** 1 Edward S. Rogers Sr. Department of Electrical and Computer Engineering, University of Toronto, Toronto, Canada; 2 Department of Computer Science, University of Toronto, Toronto, Canada; 3 Banting and Best Department of Medical Research, University of Toronto, Toronto, Canada

## Background

Cancer gene expression profiling is an indispensable tool for identifying drivers of tumor progression, identifying subtypes, and predicting clinical outcome. An outstanding challenge faced by cancer gene expression studies is the limited concordance between studies [1], driven in part by lack of statistical power [2]. Part of this lack of statistical power is due to the fact that tumor samples from some solid cancers contain between 30%-70% healthy tissue [3]. This healthy tissue contaminates tumor expression profiles and variable amounts of healthy tissue leads to increased variability between tumor expression profiles. Physical purification of these tumor samples before profiling is often not feasible.

## Materials and methods

We have developed ISOpure [4], a computational method to purify tumor gene expression profiles using reference samples of healthy tissue to model the contribution of healthy tissue. For every tumor expression profile in the input, ISOpure estimates the percentage of cancerous tissue and outputs a purified cancer expression profile from which the impact of healthy tissue has been removed. We verified our purification procedure by measuring the performance of expression-based predictive models of patient outcome in cancer, using either the original or ISOpure-purified expression profiles. We predicted extraprostatic extension (EPE) in 89 prostate tumor samples and patient survival for a set of 443 lung cancer patients.

## Results and conclusions

**Figure 1:**
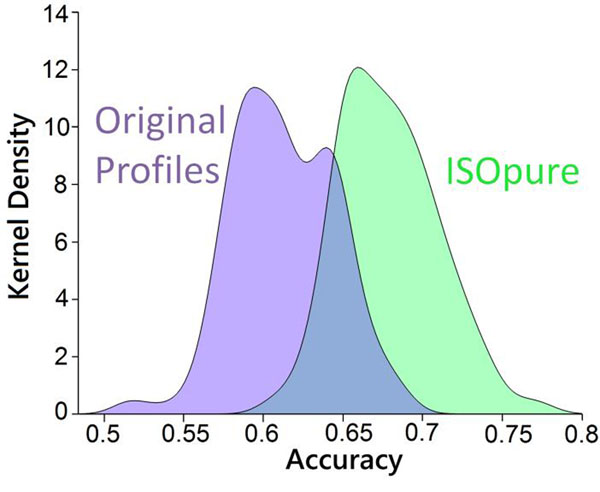
Density estimate of EPE classifier accuracy using purified and original expression profiles.

Purified expression profiles showed significant improvements in prognostic model performance. 93% of the EPE classifiers constructed using the purified profiles had higher accuracy on held-out data in cross-validation than the matching classifier trained using the original expression data (p = 1.58x10-77), with an average improvement of 11% in performance (Fig. 1). For lung cancer, the prognostic model based on the purified profiles improved hazard modeling by 39% over the model based on the unpurified profiles (p = 0.016).

We have demonstrated that ISOpure improves our ability to predict patient phenotype based on gene expression, and expect to see similar improvements for other cancer gene expression analyses such as subtype identification and classification. We are currently generating a compendium of purified gene expression profiles from 1600 tumor samples representing 15 different types of solid cancer using archival data from GEO. We are excited to work with the community at large to generate a resource of computationally purified cancer datasets, in order to facilitate more accurate analysis of cancer gene expression.
